# Advanced Machine Learning Techniques for Corrosion Rate Estimation and Prediction in Industrial Cooling Water Pipelines

**DOI:** 10.3390/s24113564

**Published:** 2024-05-31

**Authors:** Desiree Ruiz, Abraham Casas, Cesar Adolfo Escobar, Alejandro Perez, Veronica Gonzalez

**Affiliations:** Centro Tecnológico de Componentes-CTC, Scientific and Technological Park of Cantabria (PCTCAN), 39011 Santander, Spain; druizp@centrotecnologicoctc.com (D.R.); cescobar@centrotecnologicoctc.com (C.A.E.); vgonzalez@centrotecnologicoctc.com (V.G.)

**Keywords:** corrosion rate prediction, industrial cooling water pipelines, machine learning, neural networks, data preprocessing

## Abstract

This paper presents the results of a study on data preprocessing and modeling for predicting corrosion in water pipelines of a steel industrial plant. The use case is a cooling circuit consisting of both direct and indirect cooling. In the direct cooling circuit, water comes into direct contact with the product, whereas in the indirect one, it does not. In this study, advanced machine learning techniques, such as extreme gradient boosting and deep neural networks, have been employed for two distinct applications. Firstly, a virtual sensor was created to estimate the corrosion rate based on influencing process variables, such as pH and temperature. Secondly, a predictive tool was designed to foresee the future evolution of the corrosion rate, considering past values of both influencing variables and the corrosion rate. The results show that the most suitable algorithm for the virtual sensor approach is the dense neural network, with MAPE values of (25 ± 4)% and (11 ± 4)% for the direct and indirect circuits, respectively. In contrast, different results are obtained for the two circuits when following the predictive tool approach. For the primary circuit, the convolutional neural network yields the best results, with MAPE = 4% on the testing set, whereas for the secondary circuit, the LSTM recurrent network shows the highest prediction accuracy, with MAPE = 9%. In general, models employing temporal windows have emerged as more suitable for corrosion prediction, with model performance significantly improving with a larger dataset.

## 1. Introduction

Corrosion refers to a process wherein a solid substance, specifically a metal, undergoes alteration through a chemical reaction with oxygen. It entails the gradual deterioration of the material, characterized by its consumption or erosion over time. In a broader sense, it can be understood as the general tendency of materials to seek a state of greater stability or lower internal energy. The study and mitigation of corrosion are critical, as it has significant repercussions at economic, safety, and environmental levels.

During the last century, several studies have focused on the national economic impact of corrosion. Through different approaches, all have determined costs equivalent to about 3–4% of each nation’s gross domestic product (GDP). In the case of Spain, this amounts to over EUR 40 billion in the year 2022 [1,2]. These economic losses have a negative impact on the competitiveness of industries and, consequently, on the country’s economic growth. The implementation of effective corrosion models and scientific management could reduce expenditure by 25–30% [3].

Nevertheless, implications for safety and the environment are even more significant. The weakening of metallic structures due to corrosion can cause structural failures that endanger people’s lives. Furthermore, the corrosion of pipelines and equipment can result in spills of hazardous and polluting substances, leading to detrimental ecological and human effects.

Another aspect of concern is the limit of resources on our planet. At present, recycling has become a common practice, and society has attained a heightened awareness. The recycling of metal products has gained recognition for its pivotal role in preserving these finite resources. However, corroded parts of metals cannot be recycled as they would affect the quality of the material. Hence, it is fundamental to understand the progression of corrosion so that when a component reaches critical corrosion levels, it can be replaced with a new one, allowing the old component to be maximally reused [4].

For all these reasons, implementing strategies to monitor and control corrosion rates on infrastructures becomes essential.

One of the primary areas where corrosion commonly occurs is in water-cooling circuits, which are present in numerous industrial sectors (such as steel, chemical, energy, and food industries) due to their advantages in terms of efficiency and temperature control capability. In this case, the corrosion rate depends on the water and system metal characteristics. Nevertheless, water-cooling systems present other disadvantages:

- Scaling, caused by the precipitation of insoluble compounds at high temperatures, such as calcium carbonate. Scaling interferes with heat transfer and reduces flow.
- Fouling and biofouling, resulting from the settlement of suspended solids, the formation of corrosion products, and the growth of microbial masses.

Before artificial intelligence broke into pipeline corrosion management, it was not possible to estimate the future corrosion status of the system based on the fluid properties and industrial operations related to pipelines. Some authors attempted to apply classical statistical models, such as ARIMA, to forecast corrosion rates [5]. However, there is limited literature on this subject, mainly because these models have not succeeded in the sector, as they are unable to consider the impact of other variables and accurately predict changes when conditions are altered.

The introduction of machine learning into corrosion research has enabled the accurate estimation of corrosion evolution in distinct environments. Numerous authors have recently explored this topic, testing the performance of different types of algorithms. The emergence of artificial intelligence methods in structural engineering in recent years has brought forth promising opportunities to revolutionize the corrosion monitoring process in the oil and gas industry [6,7]. With greater generality, some studies discuss the role of data-driven approaches and machine learning (ML) in advancing materials science, particularly through the Materials Genome Initiative [8,9]. While ML has significantly impacted energy storage applications, its application in corrosion research is still emerging. Corrosion data are typically complex and challenging for traditional methods, but ML offers a flexible, cost-effective solution for improving predictions.

The use case [10] involved creating a detailed, structured database to support a review of ML in corrosion prediction. This work concludes that machine learning (ML) holds significant potential for improving corrosion prediction, offering more accurate and cost-effective solutions compared to traditional methods. It highlights the challenges posed by complex and heterogeneous corrosion data and the necessity for standardized evaluation metrics and procedures.

It is worth noting that in the literature, two distinct approaches to addressing the problem have been identified: considering the time variable and omitting it. The first approach involves treating the data as temporal sequences, whereas the second considers instantaneous corrosion as a function solely of external variables. For instance, the corrosion rate of steel has been predicted under marine conditions, only considering the effect of temperature, dissolved oxygen, salinity, pH, and oxidation–reduction potential, employing support vector machines for regression (SVR) and dense neural networks [11]. In this study, superior results are observed with the first model compared to the second. This is intriguing since neural networks typically deliver more favorable outcomes than SVR. They can deal with non-linearities and identify the slight changes in the input variables, a capability that SVR, in contrast, lacks [12]. The research work [13] presents a novel method for identifying corrosion types using electrochemical noise (EN) signals with artificial neural networks (ANNs) and finds that a support vector machine (SVM) outperforms backpropagation (BP) in accuracy for this purpose.

Other authors have highlighted the performance of dense neural networks and tree-type algorithms when estimating the outdoor atmospheric corrosion rate of low-alloy steel, considering time as part of the set of input variables for models [14]. Nevertheless, treating time as a regular variable may not be the most effective approach. There exist specific neural networks that have emerged to handle temporal dependencies, such as long short-term memory (LSTM) recurrent networks. In [15], the corrosion in alloys is examined through different machine learning and deep learning methods, leading to the conclusion that LSTM models are the most appropriate, as they can capture the inherent time-series relationship among data samples and could be employed in predicting long-term evolution.

Convolutional neural networks (CNNs) represent another type of neural network, designed specifically for image processing. Nonetheless, they can also be applied to numerical inputs, as an image essentially translates into a matrix of integers. Many studies have utilized CNNs to study the advancement of corrosion using images [16,17]. Yet, it is less common to encounter works exploring the application of CNNs to numerical corrosion data, despite the existence of some studies such as [18]. In other time-dependent problems, like those related to energy generation or consumption, the use of these models is more widespread, since this type of network can extract intricate patterns and dependencies from the data, as well as LSTMs [19,20].

As it is aimed in [21], it is worth developing more accurate prediction models powered by AI methodologies, facilitating timely maintenance interventions and mitigating unexpected failures. Furthermore, the corrosion rate is the most important kinetic parameter for predicting and modeling the service-life performance of reinforced concrete structures [22,23,24].

In line with the findings previously discussed, this work focuses on the development of a predictive corrosion model based on machine learning and deep learning techniques. Implementing a strategy of this nature offers numerous benefits to businesses as it results in cost savings in maintenance. It not only can help extend the lifespan of structures and equipment but also can prevent damage or production reductions by enabling proactive or corrective actions when necessary, optimizing performance. In the context of an industrial plant, this type of tool constitutes the cornerstone of a corrosion management system 4.0.

The selected case of study for this work is the cooling circuit of a local steelmaking industrial plant. This system comprises both direct and indirect cooling, along with a water treatment station for reusing within the same process to minimize water consumption. In the direct cooling circuit, commonly referred to as the primary circuit, the water comes into direct contact with the steel product, whereas in the indirect cooling circuit, known as the secondary circuit, this does not occur. All pipelines in this system are made of carbon steel, and several sensors were installed within them to motorize the parameters that potentially can have the greatest impact on the corrosion rate.

Based on the measurements collected by these sensors, several models have been developed for two distinct applications. On one hand, a virtual sensor has been engineered to estimate the corrosion rate through the online-measured variables in the waters. On the other hand, a predictive tool has been created to forecast the future evolution of corrosion, considering prior values of both the measured variables in the waters and the corrosion rate. In this second application, it is necessary to establish a time window indicating how many past instances will be used to generate predictions and how many future instances will be forecasted.

Different algorithms have been tested for each approach. For the virtual sensor, multiple linear regression, extreme gradient boosting, and dense neural networks have been evaluated. The first is probably too simple to accurately model corrosion behavior, but it serves as a baseline model. The second one has been proposed since several authors in the literature have highlighted the performance of tree-type algorithms for this task. A single decision tree is a weak model; nevertheless, extreme gradient boosting (XGBoost) creates trees iteratively, achieving a reduction in error progressively. Additionally, dense neural networks have been tested for the same reason, as they have proven to precisely handle complex relationships and to detect slight changes in data.

Conversely, for the predictive tool with a temporal window, more sophisticated neural networks have been employed. Convolutional neural networks (CNNs) and long short-term memory (LSTM) neural networks have been selected to extract underlying features of data, considering past information (in time) when processing new information. Other authors have already demonstrated that LSTM networks can efficiently handle temporal sequences; however, this study will be pioneering in the use of CNNs to predict the corrosion rate based on historical numerical values.

## 2. Methodology

This section provides a technical overview of the monitored variables and machine learning models employed in this study. Initially, the monitored variables are classified as input features and response variables.

Subsequently, the models utilized for virtual sensor approximation and predictive analytics are presented. The section will cover the mathematical foundations and algorithms underlying these models, emphasizing their applicability in simulating sensor outputs and forecasting trends over a time horizon. The calibration and validation processes of these models ensuring their robustness and accuracy in real-world scenarios will be also discussed.

### 2.1. Monitored Variables

The variables outlined in Table 1 were monitored using commercial sensors. It should be noted that the data were provided by an industrial partner; however, due to confidentiality reasons, further details about the sensors have not been disclosed. These variables were monitored over a two-year period from January 2021 to January 2023.

### 2.2. Machine Learning Models

#### 2.2.1. Virtual Corrosion Sensor

Multiple linear regression is a supervised learning algorithm in which the dependent variable to be estimated y can be expressed as a linear function of the variables x that have an impact on it.

**Figure 1:**
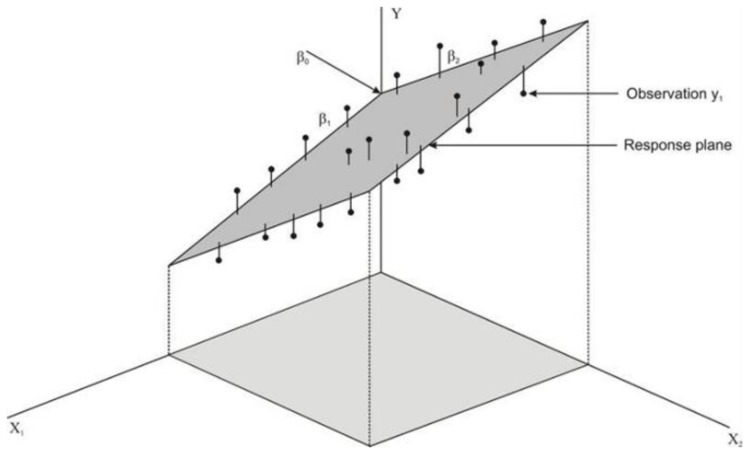
Fit of the multiple linear regression model (plane) to a dataset represented in a three-dimensional space [25].

The coefficients for x variables are determined using the method of least squares. This approach involves finding the n-dimensional space that provides the best fit for the given data points. To illustrate, when dealing with two independent variables, the data points may be visualized in a three-dimensional space $\left(x_{1},x_{2},y\right)$. The multiple linear regression model then identifies the optimal plane that encapsulates these data points, as Figure 1 shows.

#### 2.2.2. Extreme Gradient Boosting (XGBoost)

**Figure 2:**
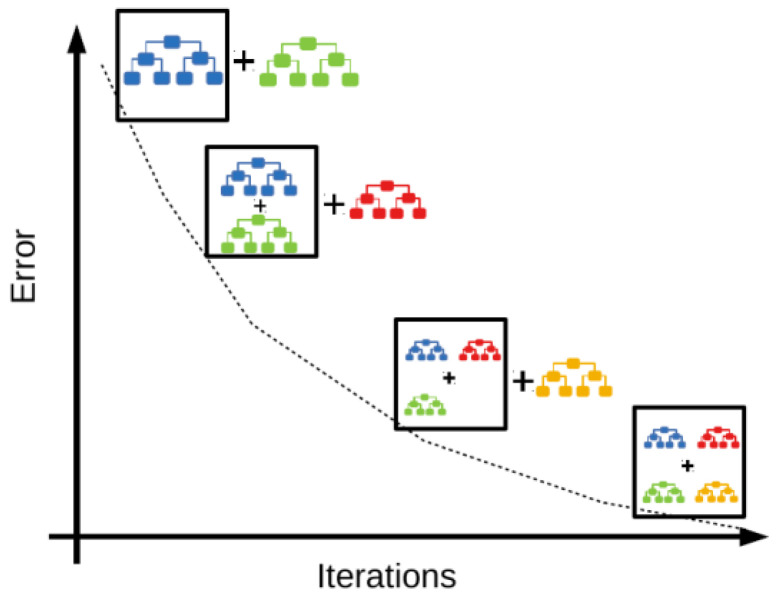
Representation of the iterative process carried out in extreme gradient boosting [33]. Each color represents a different decision tree model.

Extreme gradient boosting has become an extremely popular machine learning algorithm in recent years [26,27,28], used for both classification and regression problems. It is an enhancement of the gradient boosting algorithm [29,30,31,32], which combines multiple weak models (decision trees) and trains them sequentially to improve their performance. Each new model is created to correct the errors made by the previous models (Figure 2), allowing the algorithm to adapt and learn iteratively, reducing the error in each iteration.

XGBoost comes with a multitude of hyperparameters that must be fine-tuned for optimal model performance. An inadequate configuration of these parameters may lead to overfitting, where the model fits the training data so closely that it struggles to generalize and make accurate predictions on new data.

There exist several techniques for finding optimal values for hyperparameters, with Bayesian optimization being the most suitable for XGBoost. This technique employs a probabilistic approach to systematically search for the best parameter values. To achieve this, it constructs an objective function that models the algorithm’s performance based on these parameters and employs the obtained information to efficiently explore the best configurations. Nevertheless, this method requires specific ranges to be provided for searching these hyperparameters. If the ranges are too broad, the algorithm takes a considerable amount of time to converge (hours).

Given that optimizing the complete set of XGBoost hyperparameters would be time-consuming and that some of them are directly derived from others, only the crucial parameters in the model architecture have been optimized through Bayesian optimization. Table 2 and Table 3 collect the optimized hyperparameters and their search ranges for primary and secondary circuits, respectively.

It is worth mentioning that for the remaining parameters of XGBoost not included in Table 2 and Table 3, their default values have been preserved.

#### 2.2.3. Artificial Neural Networks (ANNs)

Artificial neural networks are mathematical models inspired by the biological neural networks that constitute the human brain [34]. They are employed in tasks such as classification, regression, pattern recognition, and decision-making. Consisting of several processing units called ‘neurons’ or ‘nodes’, these networks are arranged in different layers and interconnected [35]. Each neuron processes incoming information through an activation function, deciding whether to transmit this information to the neurons in the next layer. The network can be configured in infinite ways. It must contain at least one input layer (capturing the initial data) and one output layer (generating predictions). Nevertheless, any number of intermediate (hidden) layers can be introduced between them. Additionally, the number of neurons per layer is variable and can be configured.

**Figure 3:**
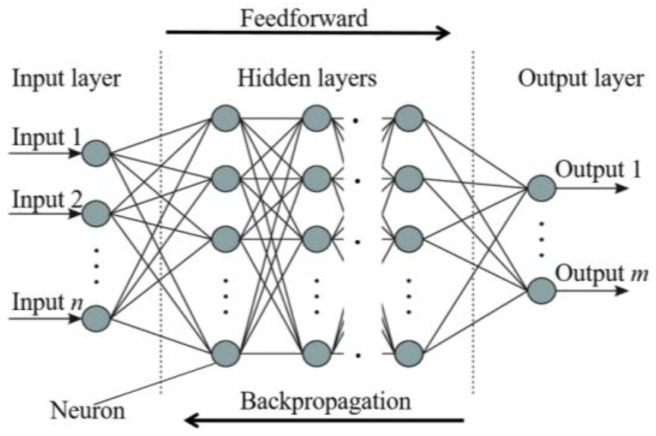
Representation of dense neural network (DNN) architecture and learning process [39].

There are various types of neural networks: dense [36], convolutional [37], recurrent [38], etc. But the learning process in these models is similar. As Figure 3 displays, it consists of two phases: feedforward, in which the input data propagate through the neural network and a prediction is made, and backpropagation, in which the prediction is compared with the real value and weights of connections between neurons are modified to reduce the error. Optimization techniques are employed to minimize this error efficiently.

The term ‘dense’ is used to describe networks with at least one hidden layer, where each neuron is connected to all neurons in the subsequent layers.

At times, ANNs can become so complex that they overfit the training data. To address this, regularization techniques like Lasso and Ridge, or the application of ‘dropout’, which deactivates a percentage of neurons within a layer, can be employed.

Furthermore, there is not an established method for determining the optimal neural network for a given problem. This remains an open research field. Refining the network architecture can only be achieved through a process of trial and error.

**Figure 4:**
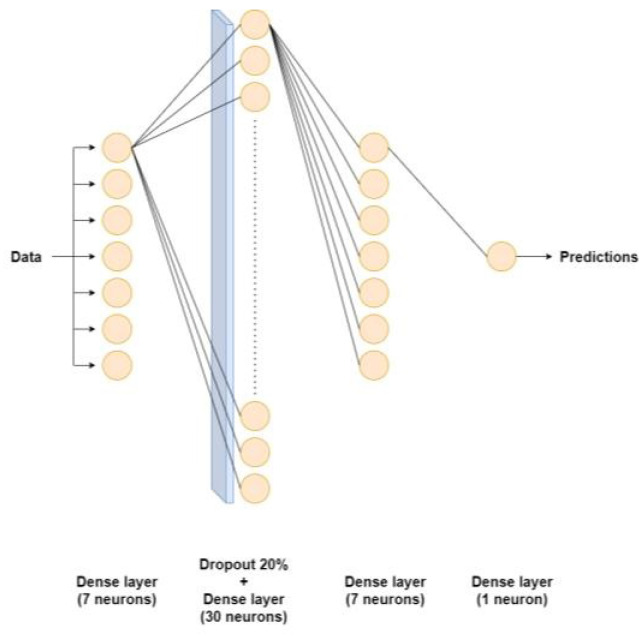
Dense neural network applied to the primary circuit. For better visualization, only the connections of the first neuron of each dense layer are represented.

**Figure 5:**
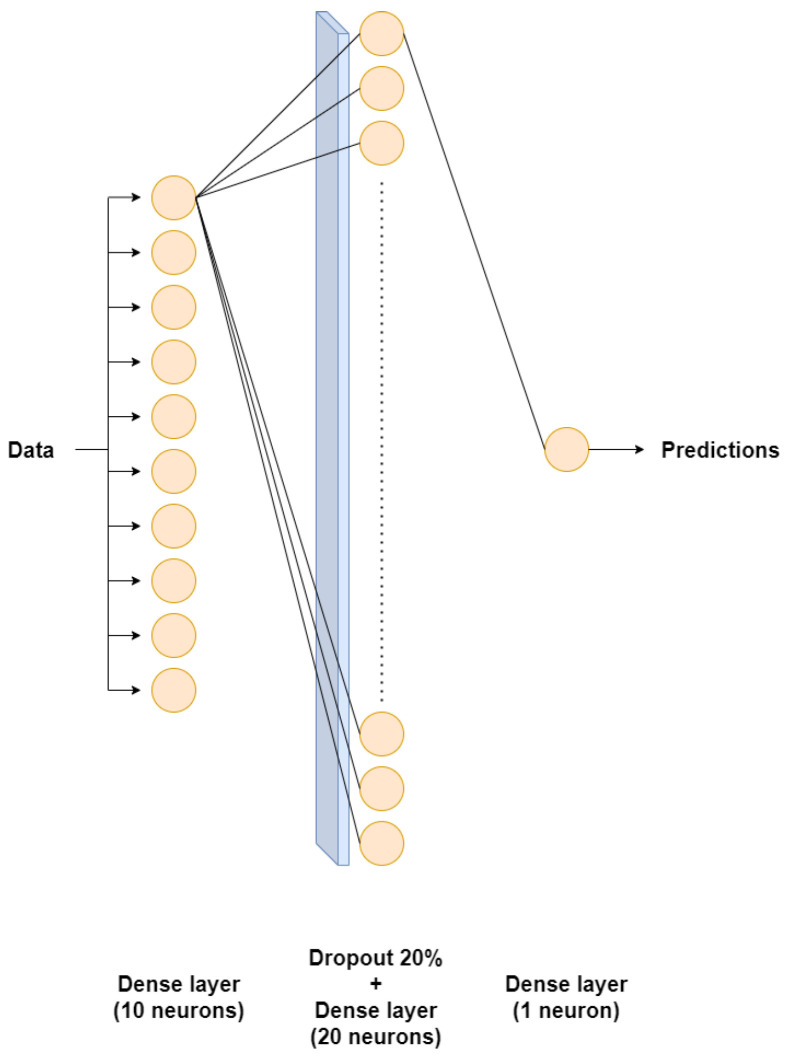
Dense neural network applied to the secondary circuit. For better visualization, only the connections of the first neuron of each dense layer are represented.

Table 4 and Table 5 delineate the architectures of dense neural networks employed in modeling the primary and secondary circuits respectively. Moreover, these architectural representations are visually depicted in Figure 4 and Figure 5 for enhanced clarity.

The network described in Table 4 has been trained with a batch size of 20 data points for 300 epochs. In addition, the mean absolute percentage error (MAPE) has been used as the loss function, and the Adaptive Moment Estimation (Adam) algorithm has been employed as the optimizer.

The network described in Table 5 has been trained with a batch size of 8 data points for 300 epochs. In addition, the mean absolute percentage error (MAPE) has been used as the loss function, and the Adaptive Moment Estimation (Adam) algorithm has been employed as the optimizer.

##### Convolutional Neural Networks (CNNs)

CNNs, as a type of ANN, are commonly used in computer vision for image classification [40,41], although they can also be applied to regression problems [42]. The cornerstone of such models is the convolutional layer, designed to extract features from the inputs. To achieve this, it applies multiple filters (also known as kernels), each performing a distinct convolution operation [43], followed by an activation function that introduces non-linearity to the model. The output of this layer is a set of feature maps (one map per filter).

**Figure 6:**
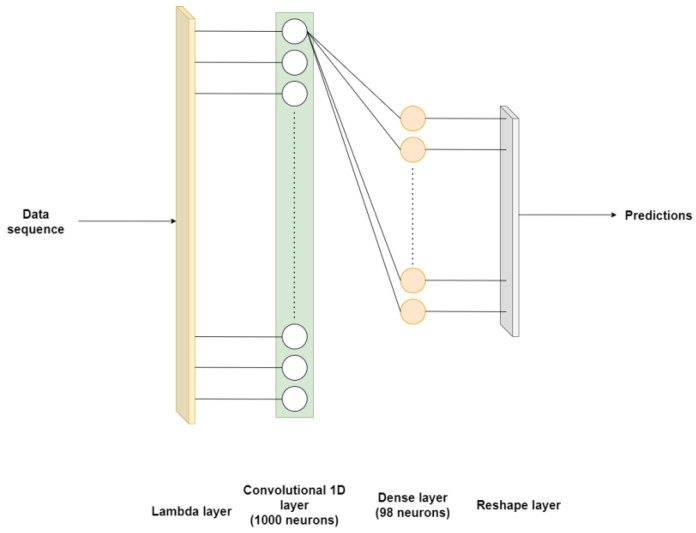
Convolutional neural network applied to the primary circuit. For better visualization, only the connections of the first neuron of the convolutional layer are represented.

Table 6 and Table 7 explain the architectures of the CNN models applied to primary and secondary circuits, respectively, and Figure 6 represents the first visually.

It is worth noting that the CNN described in Table 6 has been trained with a batch size of 8 samples for 500 epochs, and an early stopping method has been implemented to conclude training in case there is no significant improvement after 20 epochs. In addition, the mean squared error (MSE) has been used as the loss function, and the ‘Adaptive Moment Estimation’ (Adam) algorithm has been employed as the optimizer.

In contrast, the CNN described in Table 7 has been trained with a batch size of one sample for 300 epochs, due to the small amount of accurate data for the secondary circuit (which will be discussed later). Similarly, an early stopping method has been implemented to conclude training in case there is no significant improvement after 4 epochs. The loss function and optimizer used in this case are the same as those mentioned previously, namely MSE and Adam.

##### Recurrent Neural Networks (RNNs)

Recurrent neural networks and, in particular, long short-term memory models [44] are characterized by their capability to preserve information over time. They find frequent application in tasks involving sequential data, such as machine translation or natural language processing [45,46], where understanding temporal context is essential. The functionality of these models relies on the notion of ‘memory cells’, which retain past information and integrate it with current data to produce an output, continually updating their state. Throughout the training process, the weights of feedback connections are fine-tuned using backpropagation algorithms over time.

A challenge inherent to these networks is the phenomenon known as ‘gradient vanishing’ [47], which describes a situation in which gradients diminish exponentially as they propagate backward. This complicates the weight update process and adversely affects long-term learning. In response to this issue, long short-term memory (LSTM) networks have been introduced as a specialized form of recurrent networks, with a more sophisticated internal structure. LSTMs incorporate distinct gates (input, output, and forget gates [48]) to control, update, and retain information stored within the cell over extended periods in a more effective way.

**Figure 7:**
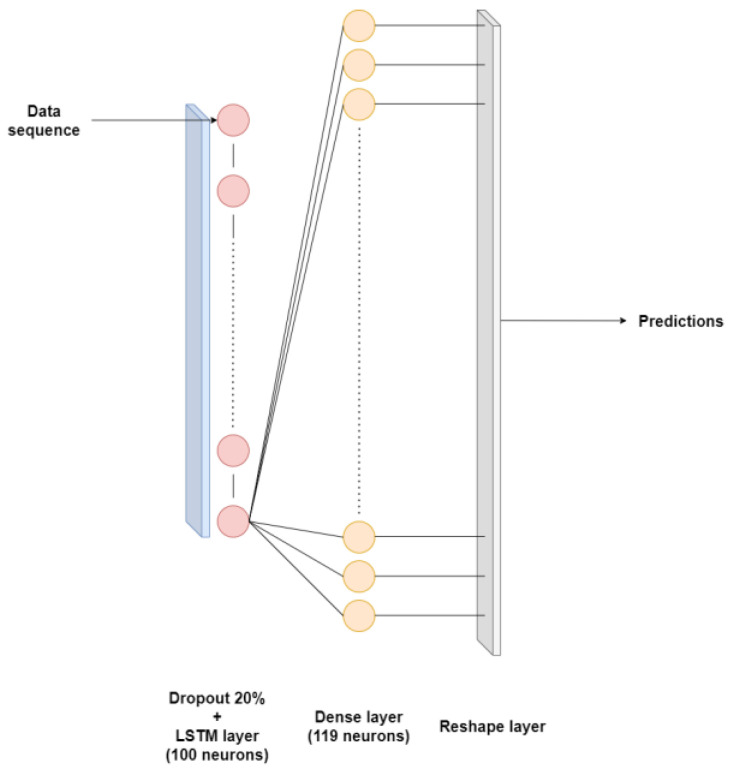
Long short-term memory neural network applied to secondary circuit.

Table 8 and Table 9 delineate the architectural specifications of the LSTM models employed in the modeling of primary and secondary circuits, respectively. Furthermore, Figure 7 illustrates the architecture corresponding to Table 9.

The training characteristics of the models described in Table 8 and Table 9 are identical to those previously described for the CNNs for each circuit.

## 3. Results and Discussion

### 3.1. Data Analysis and Preparation

A crucial step in the development of a machine learning model entails carrying out a preliminary analysis of the available data, which is necessary to safeguard against the introduction of erroneous values into the model and to understand the relationships between variables. It is important to note that data may be inaccurate and that there can be missing information. Therefore, data must be preprocessed to ensure the optimal performance of a machine learning algorithm.

#### 3.1.1. Statistical Analysis

A thorough exploration of the available data has been conducted. This has allowed for the identification of normal operational conditions ranges and the detection of anomalous values, as well as their frequency.

The abscissa axis limits for all histograms in this section have been set based on the minimum and maximum values of the represented variable.

**Figure 8:**
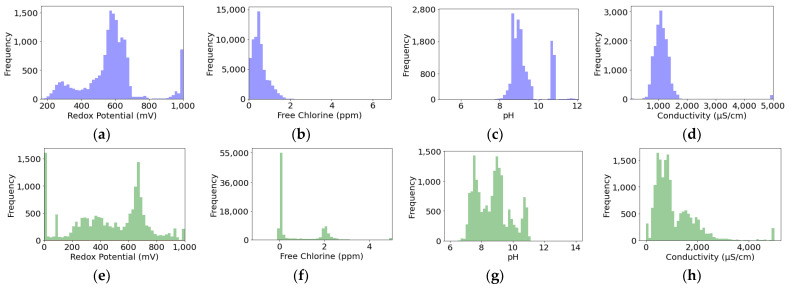
Water property histograms. In blue, for the primary circuit. In green, for the secondary circuit. Subfigures (**a**,**e**) represent redox potential (mV), (**b**,**f**) represent free chlorine (ppm), (**c**,**g**) represent pH, and (**d**,**h**) represent conductivity (µS/cm).

In Figure 8, it can be observed that most variables fall within acceptable ranges, starting from zero, except for in Figure 8f, where it is noted that there are negative values of free chlorine that have been recorded on the secondary circuit. However, it cannot be discerned what the quantity of anomalous values is.

Furthermore, in the graphs related to the secondary circuit, Figure 8e–h, bimodal distributions are noticeable, possibly stemming from the intermittent nature of its operation. Consequently, certain data points correspond to active periods, while others do not. This behavior is also observed in the redox potential and pH of the primary circuit. Nevertheless, this circuit is never turned off; therefore, further investigation into the direct cooling process should be conducted.

**Figure 9:**
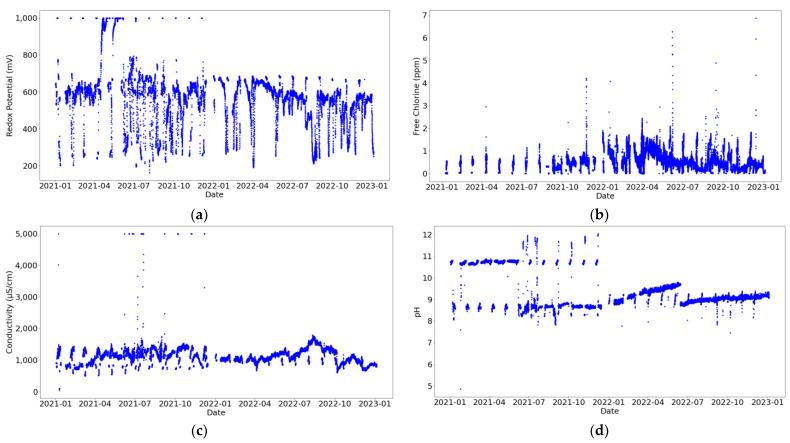
Temporal evolution of water properties in the primary circuit. Variables represented are (**a**) redox potential (mV), (**b**) free chlorine (ppm), (**c**) pH, and (**d**) conductivity (µS/cm).

**Figure 10:**
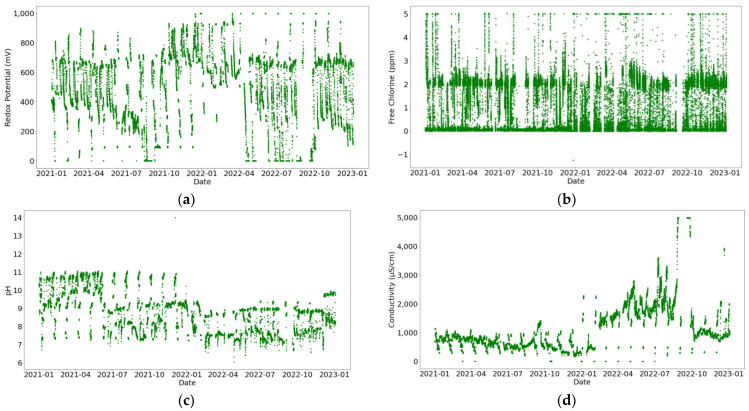
Temporal evolution of water properties in the secondary circuit. Variables represented are (**a**) redox potential (mV), (**b**) free chlorine (ppm), (**c**) pH, and (**d**) conductivity (µS/cm).

The water measurements represented in Figure 9 and Figure 10 display substantial variability, especially for the redox potential due to the high sensitivity of this sensor. These fluctuations are not caused by signal noise, but rather by alterations in the water’s chemical composition resulting from water treatment (such as the introduction of corrosion inhibitors). This aims to improve cooling system functionality. Consequently, out-of-range data points are not excluded as they represent real states of the systems. In Figure 10b, it can be observed that there is only one anomalous value of conductivity (−1 µS/cm), which may be attributed to a measurement error or sensor connection issue.

Furthermore, it can be observed that the redox potential and pH sensors installed in both circuits saturate at 1000 mV and 5000 µS/cm, as shown in Figure 9 and Figure 10a,d, respectively. Moreover, the free chlorine sensor placed in the secondary circuit also saturates at 5 ppm, as shown in Figure 10b.

**Figure 11:**
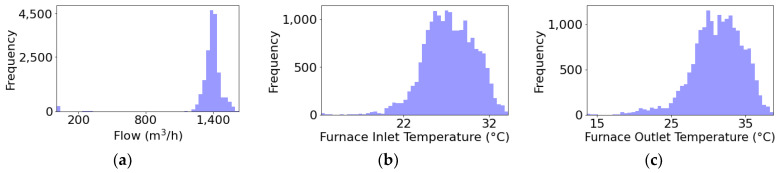
Pipeline characteristics histogram for the primary circuit. Variables represented are (**a**) flow (m^3^/h), (**b**) furnace inlet temperature (°C), and (**c**) furnace outlet temperature (°C).

**Figure 12:**
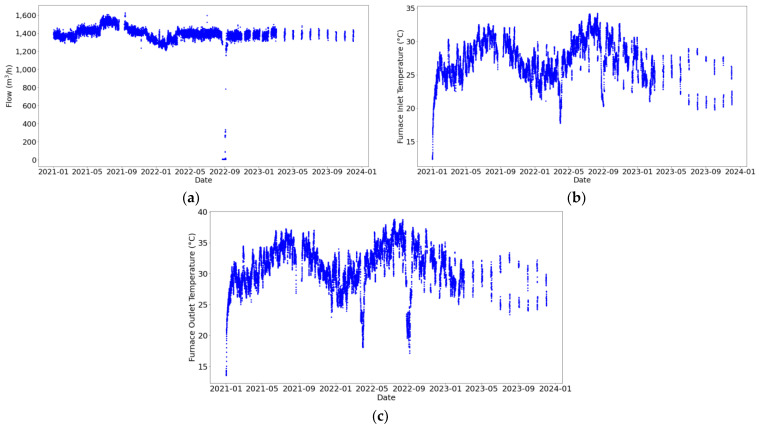
Temporal evolution of pipeline characteristics in the primary circuit. Variables represented are (**a**) flow (m^3^/h), (**b**) furnace inlet temperature (°C), and (**c**) furnace outlet temperature (°C).

In Figure 11b,c, it can be observed that the temperature distribution at the furnace outlet is higher than that at the inlet, as expected. Additionally, a greater variability in temperatures is noted compared to the flow, which remains relatively constant. This indicates that the process is continuous even though there is a noteworthy drop in flow around September 2022 (Figure 12a), when it decreases to approximately 6 m^3^/h.

**Figure 13:**
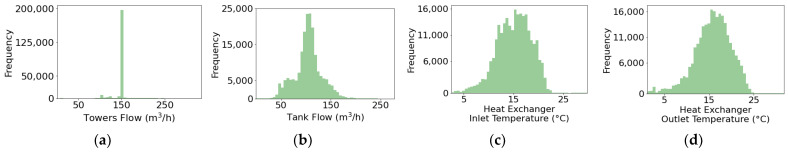
Pipeline characteristic histograms for the secondary circuit. Variables represented are (**a**) tower flow (m^3^/h), (**b**) tank flow (°C), (**c**) heat exchanger inlet temperature (°C), and (**d**) heat exchanger outlet temperature (°C).

**Figure 14:**
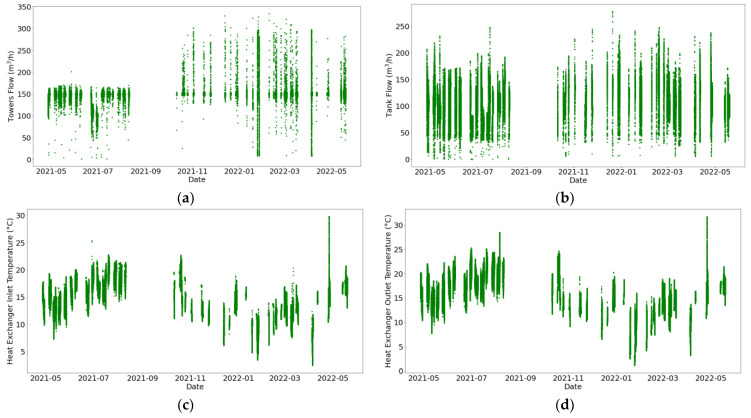
Temporal evolution of pipeline characteristics in the secondary circuit. Variables represented are (**a**) tower flow (m^3^/h), (**b**) tank flow (°C), (**c**) heat exchanger inlet temperature (°C), and (**d**) heat exchanger outlet temperature (°C).

As Figure 13 displays, there are no anomalous values of the flows and temperatures in the secondary circuit. Nonetheless, it can be seen in Figure 14 that numerous periods exhibit lack of measurements. Furthermore, fluctuations are more pronounced in the secondary circuit, although both circuits exhibit acceptable variations in flow and temperature, considering the experimental conditions. This is attributed to changes in the secondary circuit state (active/inactive); thus, it is indicated as a discontinuous process.

**Figure 15:**
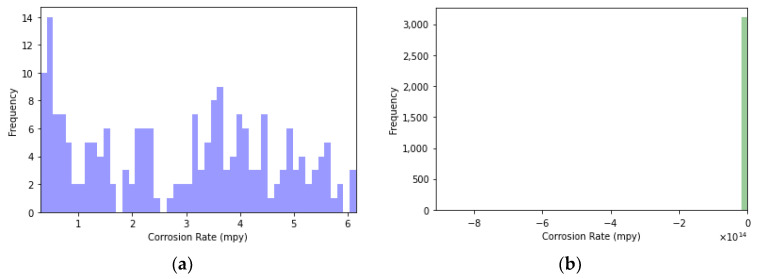
Corrosion rate histograms: (**a**) at the primary circuit and (**b**) at the secondary circuit.

It can be observed in Figure 15 that the measures taken by the corrosion sensor in the primary circuit fall within acceptable values, whereas the sensor in the secondary circuit sensor recorded values significantly outside the usual range. Upon detailed analysis of the data, it has been concluded that there is only one negative measurement of corrosion rate. When representing corrosion data over time, this value has been excluded to accurately assess the real variability of this variable in the secondary circuit.

**Figure 16:**
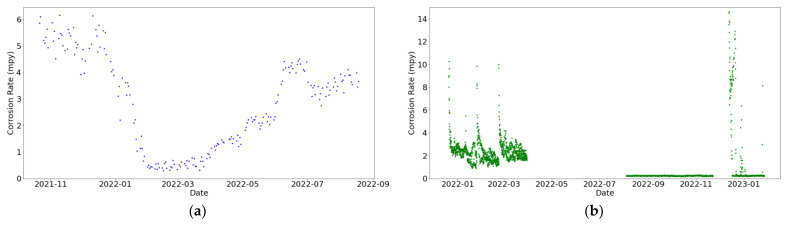
Corrosion rate over time: (**a**) at the primary circuit and (**b**) at the secondary circuit.

**Figure 17:**
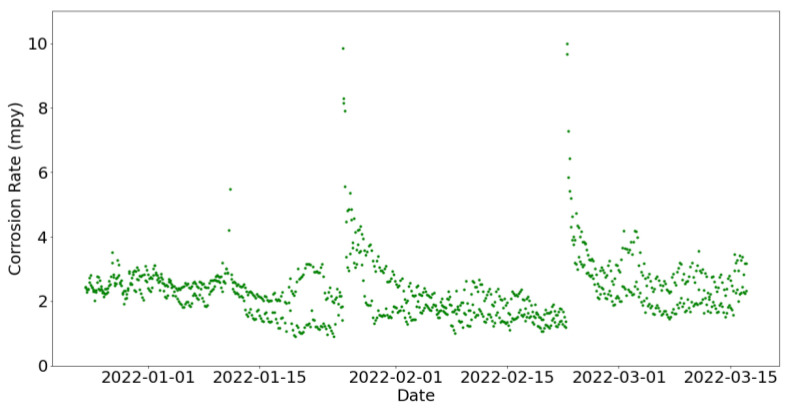
Corrosion rate at the secondary circuit during a period of proper functioning.

Figure 16a reveals the correct performance of the corrosion sensor at the primary circuit throughout the entire measurement period. In contrast, the corrosion sensor installed at the secondary circuit exhibited satisfactory functionality only during the initial months, as illustrated in Figure 16b. Subsequently, there was a pause in data acquisition, followed by an operational period during which inaccurate values were recorded. This can only be explained by a failure in the sensor; hence, only the positive data from the initial months have been considered in subsequent stages. The corrosion data selected for subsequent modeling of the secondary circuit are illustrated in Figure 17.

Two distinct peaks of corrosion rate have been primarily observed at Figure 17. It has been confirmed that these correspond to dates when the sensor was taken out for cleaning. On the day it was reinserted, it recorded a significantly high value, and in the subsequent days, consistently high values were still being registered, albeit progressively decreasing over time.

#### 3.1.2. Correlation Analysis

To address the challenge of differing data frequencies, we standardized the dataset to a daily frequency, averaging measurements for each circuit per date. This method ensures a balance between data comparability and sufficiency, enhancing the analysis for corrosion prediction. Corrosion is a phenomenon that progresses slowly, and variations within a single day are not deemed significant in this context [5].

This allowed the use of the Pearson correlation coefficient method [49] to study the statistical relationship between variables, which can provide insight into which water variables have a greater impact on corrosion. Coefficients are independent of the measurement scale of the variables and take values within the range [−1, 1]. When these coefficients approach values close to −1 or +1, a high correlation between the variables is signified.

**Figure 18:**
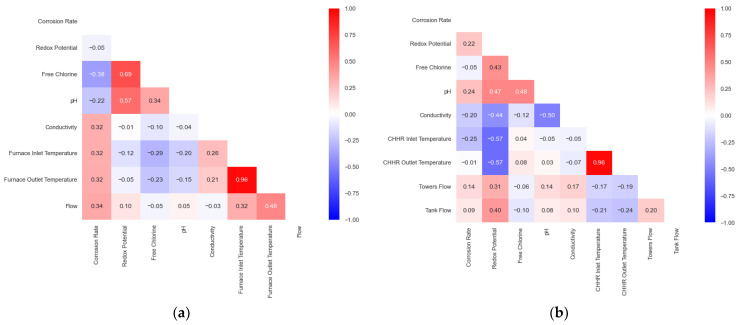
Corrosion matrix for (**a**) the primary circuit and (**b**) the secondary circuit.

It is worth noting that the coefficients shown in Figure 18 have been calculated considering only the dates for which all variables are available. Therefore, in some cases, there were more data points available for studying the correlation than in others. This is not a desired situation; thus, the results obtained from this analysis should be considered as indicative.

From Figure 18, it can be deduced that no variable exhibits a notable high correlation with the corrosion rate, neither for the primary circuit (a) nor for the secondary circuit (b). Furthermore, the results obtained for each circuit are not similar. For instance, in the primary circuit, the redox potential exhibits a low correlation with the corrosion rate, whereas in the secondary circuit, this variable shows one of the highest correlations with the corrosion rate.

#### 3.1.3. Data Preprocessing

As previously observed in the figures from Section 3.1.1, data for certain dates is unavailable, and this happens for every variable recorded. These data gaps present challenges when modeling through machine learning; hence, preprocessing techniques must be applied to the data before their incorporation into the algorithm.

One potential solution would be to remove rows of data containing gaps, but in this scenario, the frequency of these gaps is considerable, leading to a substantial loss of data. An alternative to this involves filling the gaps with probable values. The selected imputation strategy for gaps situated between numerical values was linear interpolation. Additionally, for gaps present at the earliest and latest recorded dates, the closest non-null value has been used as a substitute.

Regarding corrosion rate values, an abnormal value (negative value) was spotted during the initial analysis. We decided to remove it, as it does not significantly impact the overall data count. After preprocessing, there were 302 observations (data rows) available for the primary circuit and 83 for the secondary circuit.

### 3.2. Data Modeling

Two distinct approaches have been applied for predicting corrosion rates. Firstly, a virtual corrosion sensor aims to calculate the corrosion rate at each point, knowing the values of other variables. This approach leverages the comprehensive data on environmental and operational conditions to estimate corrosion rates accurately. Secondly, a predictive tool with a temporal window has been created for each circuit. This tool seeks to predict future corrosion rate values within a specified time window, using past values of corrosion rates and other variables. This method allows for forecasting corrosion trends and potentially planning maintenance, or preventive actions based on predicted corrosion developments. It is worth noting that different training and testing sets were used for both approaches. For the virtual sensor, as it does not consider temporal evolution, sets were randomly selected, taking approximately 80% of the total data for training and the remaining 20% for making predictions. In the case of the predictive tool, a temporal window was established to estimate the corrosion rate at several future points based on recorded variables from past instances. In this scenario, data from the most recent 14 days were used for testing, whereas previous data were employed to train the algorithm.

Furthermore, k-fold cross-validation, which is a data resampling method that is widely used to estimate the true prediction error of models and to tune model parameters, was applied to the virtual sensor. In all cases, the number of folds considered was five.

#### 3.2.1. Virtual Corrosion Sensor

The variables employed to create the virtual corrosion sensor models (multiple linear regression, XGBoost, and dense neural network), as well as the results obtained with each of them, are described below.

##### Primary Circuit

The input features for this model are as follows:

- Redox potential (mV);
- Free chlorine (ppm);
- pH;
- Conductivity (µS/cm);
- Flow (m^3^/h);
- Furnace outlet temperature (°C);
- Furnace inlet temperature (°C).

The corrosion rate in mpy (mils per year) is the output variable.

As it is impossible to graphically represent outcomes achieved through cross-validation, due to the inherent nature of the technique, results obtained with randomly chosen but identical training and testing sets in all cases are presented first. Comparing the model fitting to the training and testing data, it is possible to determine whether the model generalizes correctly or if it overfits the training data. When the latter occurs, it leads to inaccurate predictions for the test set.

Firstly, results attained with the multiple linear regression model are presented. Secondly, results achieved with the XGBoost algorithm are presented. Table 10 shows the feature importances obtained with this model. Thirdly, the results obtained with the dense neural network are presented.

**Figure 19:**
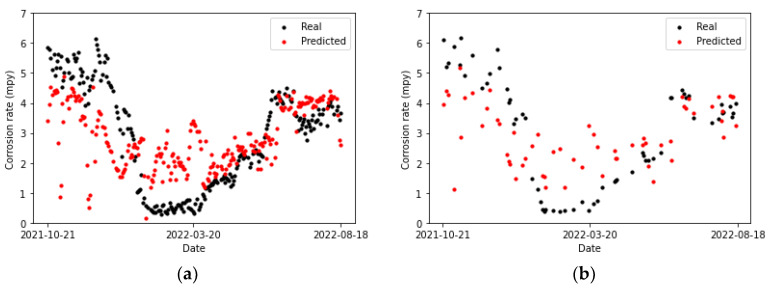
Corrosion rate at the primary circuit. The actual data recorded by the sensor are shown alongside the values predicted by the multiple linear regression model: (**a**) for the training set and (**b**) for the testing set.

**Figure 20:**
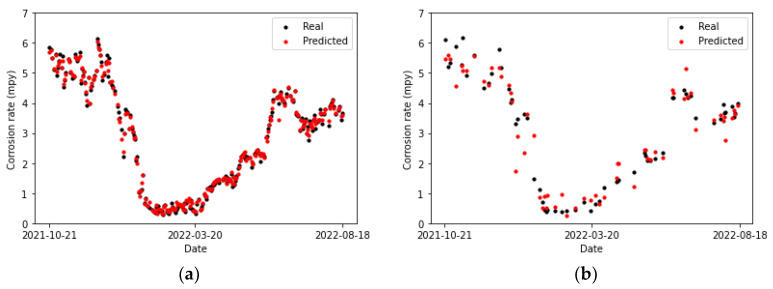
Corrosion rate at the primary circuit. The actual data recorded by the sensor are shown alongside the values predicted by the XGBoost model: (**a**) for the training set and (**b**) for the testing set.

**Figure 21:**
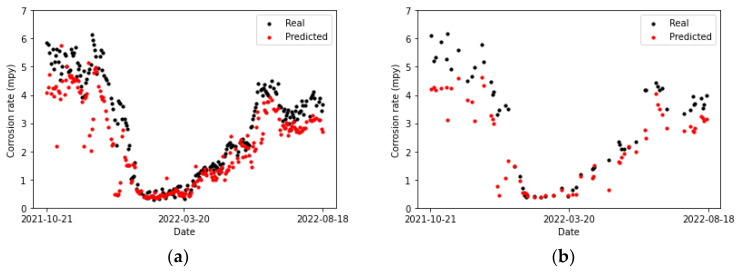
Corrosion rate at the primary circuit. The actual data recorded by the sensor are shown alongside the values predicted by the neural network model: (**a**) for the training set and (**b**) for the testing set.

Observing Figure 19, Figure 20 and Figure 21, it can be appreciated that multiple linear regression is the model that yields the poorest results: it is unable to track the trend of the actual data. Consequently, the predictions are far from it, both in the training set and the testing set. Regarding the XGBoost algorithm, it clearly overfits the training data since performance on the testing set is notably lower in comparison. This can be observed in Table 11.

**Table 10:** Feature importance obtained from XGBoost for the training set displayed in Figure 20.

Variable	Importance
pH	0.64
Furnace Inlet Temperature (°C)	0.17
Flow (m^3^/h)	0.06
Redox Potential (mV)	0.05
Free Chlorine (ppm)	0.03
Conductivity (µS/cm)	0.03
Furnace Outlet Temperature	0.02

**Table 11:** Error metrics obtained for XGBoost, tested on the sets of data displayed in Figure 20, corresponding to the primary circuit.

Error Metric	Training Set	Testing Set
MAPE (%)	4	21
MSE	0.008	0.3
RMSE	0.09	0.5
MAE	0.06	0.4

On the other hand, the metrics associated with the dense neural network model (Table 12) are similar for the training and testing sets shown in Figure 21.

The dense neural network is the model that generalizes the best: it can follow the trend of the actual values, both in the training set and the test set, although the predictions are slightly shifted below the actual values.

Finally, Table 13 compiles the results obtained through cross-validation.

A notable disparity is evident in the error values for linear regression as compared to the other two models. The mean absolute percentage error (MAPE) provided by XGBoost is consistent with that of the neural network, yet it is observable that the remaining metrics of the neural network are higher than those of XGBoost, attributable to the overfitting of XGBoost.

Table 14 shows the mean values of feature importances considering five folds in XGBoost, whereas Table 10 collects the feature importances obtained for the training set represented in Figure 20. Comparing both tables, it can be concluded that pH is the most significant variable, while redox potential is fourth in these rankings. Nevertheless, the order of importance of the remaining variables varies.

##### Secondary Circuit

The input features for this model are as follows:

- Redox potential (mV);
- Free chlorine (ppm);
- pH;
- Conductivity (µS/cm);
- Tower flow (m^3^/h);
- Tank flow (m^3^/h);
- CHHR outlet temperature (°C);
- CHHR inlet temperature (°C);
- Circuit state;
- Sensor maintenance.

Circuit state and sensor maintenance are binary variables indicating whether the secondary circuit is active and whether the sensor has been removed for cleaning, respectively. The output variable is the corrosion rate in mpy.

The performance of the multiple linear regression, XGBoost, and dense neural network models is compared, but this time for data related to the secondary circuit. Firstly, results attained with the multiple linear regression algorithm are presented. Secondly, results obtained with the XGBoost algorithm for the secondary circuit are displayed. Table 15 shows the feature importances obtained with this model. Finally, results attained with the neural network for the secondary circuit are presented.

**Figure 22:**
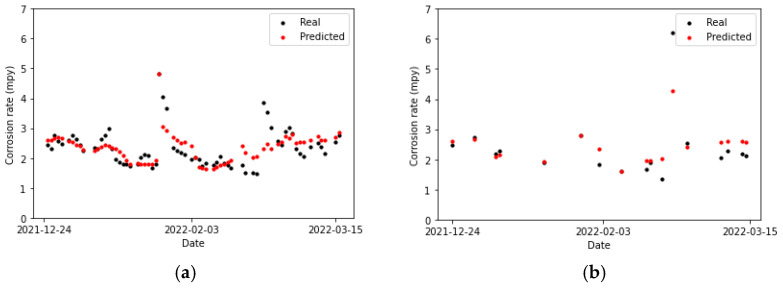
Corrosion rate at the secondary circuit. The actual data recorded by the sensor are shown alongside the values predicted by the multiple linear regression: (**a**) for the training set and (**b**) for the test set.

**Figure 23:**
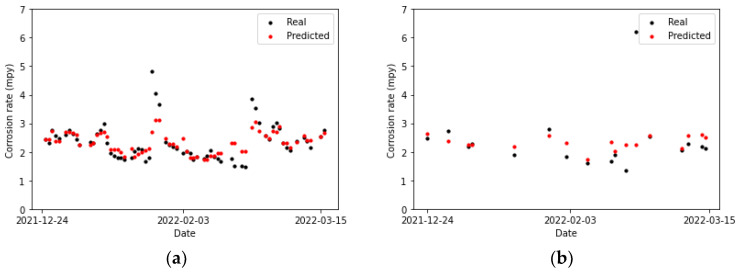
Corrosion rate at the secondary circuit. The actual data recorded by the sensor are shown alongside the values predicted by the XGBoost model: (**a**) for the training set and (**b**) for the test set.

**Figure 24:**
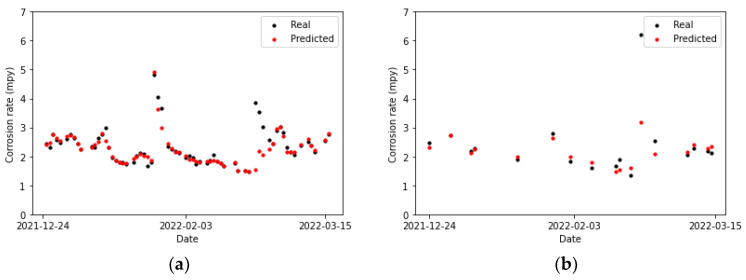
Corrosion rate at the secondary circuit. The actual data recorded by the sensor are shown alongside the values predicted by the neural network model: (**a**) for the training set and (**b**) for the testing set.

Upon examining Figure 22, Figure 23 and Figure 24, it is noted that the dense network exhibits superior adaptability to the training data compared to the other two models. In most instances, its predictions for the test data either coincide with the actual data or are positioned in immediate proximity to them. The only exception is the data point with a corrosion rate exceeding 6 mpy, for which multiple linear regression manages to predict a value that is closer, albeit approximately 2 mpy lower than the actual value. Consequently, it can be concluded that none of the models can accurately predict this point.

Finally, Table 16 compiles the outcomes obtained through cross-validation.

**Table 15:** Feature importance obtained from XGBoost for the training set displayed in Figure 23.

Variable	Importance
CHHR Outlet Temperature (°C)	0.31
Tower Flow (m^3^/h)	0.29
Tank Flow (m^3^/h)	0.11
Free Chlorine (ppm)	0.10
Potential (mV)	0.09
Conductivity (µS/cm)	0.05
CHHR Inlet Temperature (°C)	0.04
pH	0.01
Circuit State	0.00
Sensor Maintenance	0.00

The feature importance results achieved with the five-fold cross-validation XGBoost algorithm are presented in Table 17.

Comparing Table 15 and Table 17, it can be concluded that CHHR outlet temperature and tower flow remain the most important variables for XGBoost in the secondary circuit. Nevertheless, there is no variable that stands out for this importance, which differs from the results obtained for the primary circuit, where the pH importance value is appreciably greater than the rest.

Regarding model performance, significant disparity is not observed between the results provided by the three algorithms in Table 16: all their error metrics are congruent. Nonetheless, the dense neural network exhibits slightly superior performance, as in general, it yields lower error metrics compared to both multiple linear regression and the XGBoost algorithm.

It is worth mentioning that a substantial discrepancy has been found in the performance of linear regression between the primary and secondary circuits (Table 13 and Table 16). Considering that the primary difference between both circuits lies in the fact that the water in the primary circuit is in contact with the steel product, whereas the water in the secondary circuit does not, this fact could significantly affect the linearity of the process. The direct interaction of the product with water is likely to induce alterations in the water’s composition due to material loss from the product. Consequently, this may result in a notable increase in the concentration of iron ions in the water. On the other hand, the primary cooling circuit is open, meaning that the water is cooled upon contact with air and reused in the process. During this interaction, the water absorbs oxygen from the air, thus increasing the concentration of dissolved oxygen in the water. Ultimately, these concentrations of iron and oxygen ions in the water could potentially accelerate the corrosion process.

The observation that the linear model fails to yield superior results in any of the cases is in line with other researchers’ findings regarding the substantial non-linearity inherent in corrosion-related data [50]. On the other hand, the potential of ANNs, particularly DNNs, in predicting corrosion values has already been highlighted in [11,12]. In the former, MAPE precisions of 4% and 5% are achieved for specific testing sets, whereas in the latter, an average MAPE of 22% is obtained considering a large group of sets. What is truly significant is the average performance taking into account several training and testing groups, as variability is inherent to the data and, at times, the model will fit better to some sets than to others. In light of this, the present work represents an advancement as, through the technique of k-fold cross-validation, an average error of MAPE = 11% has been accomplished.

Literature reviews suggest that the employment of feature engineering techniques such as principal component analysis (PCA), which reduce the dimensionality of the problem, can enhance the corrosion rate estimation even more. Diao et al. achieved an RMSE of 0.022 in their study [51] with a determined testing set. Although the precision may vary when considering a larger number of testing sets, the fact that these authors achieved an RMSE an order of magnitude lower than those reached in the present study suggests that there is room for improvement.

#### 3.2.2. Predictive Tool for the Forthcoming 7 Days

For this approach, models based on a convolutional neural network and a long short-term memory recurrent neural network were employed. The implementation of temporal windows introduces a variation in the learning process compared to the previous scenario of the virtual sensor. In this context, the chronological order of data is taken into account when predicting subsequent moments.

##### Primary Circuit

The input features for this model are as follows:

- Two periodic functions (sine and cosine) that characterize the day of the week;
- Two periodic functions (sine and cosine) that characterize the day of the year;
- Two periodic functions (sine and cosine) that characterize the season of the year;
- Redox potential (mV);
- Free chlorine (ppm);
- pH;
- Conductivity (µS/cm);
- Flow (m^3^/h);
- Furnace outlet temperature (°C);
- Furnace inlet temperature (°C);
- Corrosion rate (mpy).

The models take the input variables over 7 consecutive days and generate from them a prediction of the corrosion rate for the next 7 consecutive days.

Table 18 collects the error metrics of the convolutional model. It is evident that all of them are lower for the testing set than for the training set. This is to be expected, as the model learns more about the complex relationships between variables as it processes new data.

**Figure 25:**
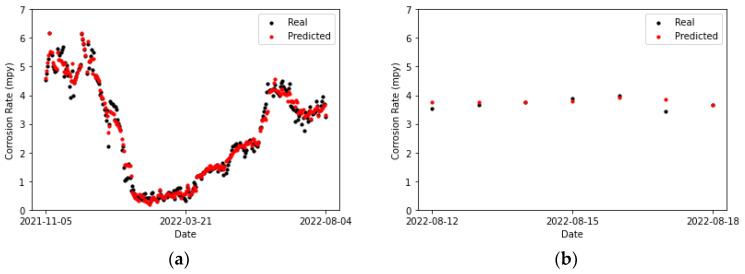
Corrosion rate at the primary circuit. The actual data recorded by the sensor are shown alongside the values predicted by the convolutional neural network model: (**a**) for the training set and (**b**) for the testing set.

**Figure 26:**
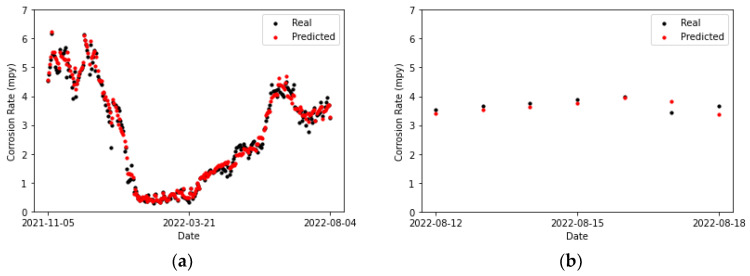
Corrosion rate at primary circuit. The actual data recorded by the sensor are shown alongside the values predicted by the LSTM network: (**a**) for the training set and (**b**) for the testing set.

In Figure 25a and Figure 26b, no discernible differences are observed between the CNN and LSTM fittings to the training set. However, the performances of these models on the testing set are a little more dissimilar. The predictions and actual values overlap for two points in Figure 26b, whereas no overlap is observed in Figure 25a. This can also be noticed by comparing Table 19, which collects the error metrics of the recurrent model, with Table 18. Overall, better results are observed for the CNN model on the primary circuit.

##### Secondary Circuit

The input features for this model are as follows:

- Two periodic functions (sine and cosine) that characterize the day of the week;
- Two periodic functions (sine and cosine) that characterize the day of the year;
- Two periodic functions (sine and cosine) that characterize the season of the year;
- Redox potential (mV);
- Free chlorine (ppm);
- pH;
- Conductivity (µS/cm);
- Tower flow (m^3^/h);
- Tank flow (m^3^/h);
- CHHR outlet temperature (°C);
- CHHR inlet temperature (°C);
- Circuit state;
- Maintenance sensor;
- Corrosion rate (mpy).

Similar to the primary circuit, the models take the input variables over 7 consecutive days and generate from them a prediction of the corrosion rate for the next 7 consecutive days. Firstly, results achieved with the convolutional neural network are presented. Table 12 explains the architecture of the model.

**Figure 27:**
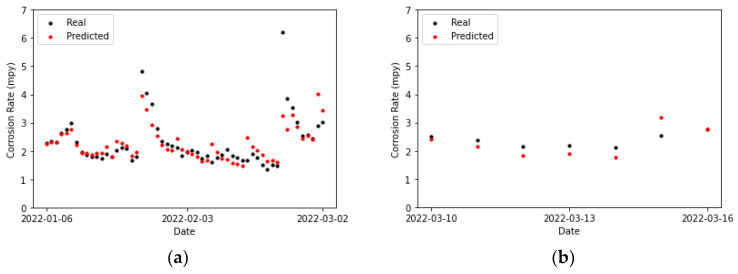
Corrosion rate at the secondary circuit. The actual data recorded by the sensor are shown alongside the values predicted by the convolutional neural network model: (**a**) for the training set and (**b**) for the testing set.

Table 20 collects the error metrics of the convolutional model. Most of them are lower for the testing set than for the training set. This is to be expected, given that algorithms employing temporal windows improve their predictions as the amount of processed time-series data grows. Furthermore, in Figure 27b, it can be observed that for two of the points (the first and the last), the actual values and those predicted by the model overlap. There is one point for which the prediction surpasses the actual value (the previous to last), and it shows the highest error. For the rest of the points, the predicted values are below the actual ones, but close to them.

Secondly, results obtained with the LSTM neural network are presented.

**Figure 28:**
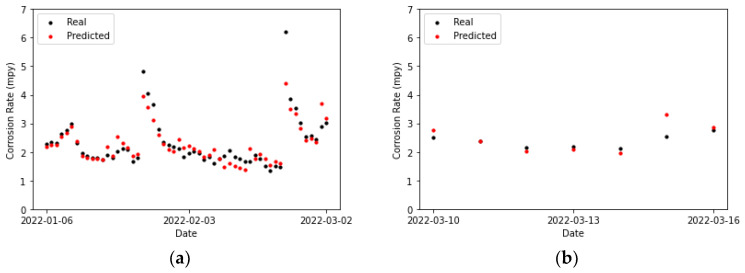
Corrosion rate at secondary circuit. The actual data recorded by the sensor are shown alongside the values predicted by the LSTM network: (**a**) for the training set and (**b**) for the testing set.

Comparing Figure 27a and Figure 28a, it can be concluded that the LSTM model fits better to the second peak than the CNN. Moreover, most of the predictions in Figure 28a are closer to the actual values compared to the convolutional network, except for the penultimate point, where the prediction of the LSTM network exhibits a slightly higher error than the convolutional network. This particular data point presents a challenge for both prediction models.

Table 21 collects the error metrics of the recurrent model. It can be noticed that lower metrics are obtained for the LSTM than for the CNN model (Table 20).

CNNs have already shown good performances for corrosion prediction; nevertheless, we lack evidence of previous studies employing such models with the same approach as in the present work. In [18], Yang et al. employ a classification approach to determine the corrosion status based on data acquired through the piezoelectric active sensing-based time reversal method, achieving an accuracy of 99.01%. In [51], Cantero-Chinchilla et al. propose a CNN to make estimations about the thickness values (minimum and mean) of corroded profiles from an ultrasonic time-series measurement. Their model surpassed traditional thickness estimation techniques in accuracy and reliability, both on synthetic and experimental data.

The former studies demonstrate the potential of CNNs in enhancing the precision of corrosion measurement tasks, each of them employing a distinct approach. This finding does not undermine the value of LSTM models but rather outlines the distinct advantages and applications of different neural network architectures depending on the dataset characteristics and the specific prediction tasks at hand.

Jiang et al. demonstrated that LSTM neural networks outperform traditional machine learning methods in predicting corrosion, specifically pitting in corrosion-resistant alloys. Their work [15], applying time-series analysis on data from 150-day saline solution immersion tests, showed that LSTMs accurately predicted corrosion potential over an additional 70 days. This indicates the superior ability of LSTM to capture the progression of corrosion over time, offering a more effective solution for predicting pitting evolution than conventional methods. On the other hand, Trung et al.’s research on the use of LSTM for atmospheric corrosion monitoring illustrates the strengths of LSTM in handling sequential data, benefiting from its ability to remember long-term dependencies [52]. Their study showcased the high accuracy of LSTM in predicting corrosion progression using data extracted through multivariate singular spectrum analysis (MSSA) from atmospheric corrosion monitoring sensors.

In summary, the best models obtained in the present study for each approach (virtual sensor and predictive tool with a temporal window) and circuit (primary and secondary) are presented in Table 22, along with the error each one yields on the test sets. It is important to recall that, for the virtual sensor, the mean error is obtained after considering various training and testing sets (applying k-fold cross-validation), whereas for the predictive tool, the error corresponds to a single test set consisting of the 14 most recent observations (days).

It is worth mentioning that the most complex algorithms, those that consider data as temporal sequences (CNN and LSTM), are also the most computationally efficient. The training duration for the DNNs was twice that of both the CNN and LSTM models, which shared an identical training time. This is mainly because they employ an early stopping method, which concludes training when there is no significant improvement in the error of predictions. This method was not implemented in the virtual sensor models, as even training for 300 epochs did not yield an error comparable to that achieved by the predictive tool. The selection of 300 epochs was deliberate since increasing this value did not result in a noteworthy enhancement. In addition, the prediction time for the testing sets is approximately 0.1 s across all methods.

Finally, in Table 22, it can be observed that for the virtual sensor approach, a higher precision is obtained for the primary circuit, whereas the opposite occurs with the predictive tool. Considering the quantity of available data in each case, it would be expected to achieve higher precision for the primary circuit in all situations. The error tends to decrease as the amount of available data grows, especially when dealing with complex algorithms such as CNNs and LSTMs. Nonetheless, in the case of DNNs, it also strongly depends on their internal architecture.

## 4. Conclusions

In this paper, two distinct approaches have been explored to model the corrosion on an industrial water-cooling system made of carbon steel. Both are built upon data concerning water properties and industrial operations related to pipelines. Nevertheless, one of them calculates corrosion based on specific input variables at a determined moment (virtual corrosion sensor), whereas the other considers the temporal evolution of those variables at previous instances (predictive tool with a temporal window).

The results illustrate that the second approach, in which deep learning algorithms have been employed, estimates the corrosion rate with less error than the first approach, which utilizes machine learning techniques. This highlights the fact that the involved variables should be considered time-independent, and that sufficiently complex models, such as convolutional neural networks and long short-term memory networks, are required to properly model the behavior of pipeline corrosion.

The window size used in these models is two weeks. This is not ideal for anticipating future corrosion early enough to implement effective mitigation measures, as corrosion is a phenomenon that progresses slowly. To achieve this, it would be necessary to have a broader historical data record, as the algorithms require more information as the complexity of the problem to be solved increases. Nevertheless, this work establishes the foundation for implementing a corrosion management system 4.0, as the predictive tool has proven to be effective in terms of both precision and computational expense. The mean absolute percentage error, which is the most ambitious metric considered in this study, is less than 10% for the testing sets considered in both primary and secondary circuits. Moreover, the training time for the CNN and LSTM algorithms is in the order of a few seconds, and the inference time is even shorter.

Furthermore, it is worth noting that, although there are few studies in the literature that employ convolutional neural networks in the study of corrosion with numerical input variables, this work has proven that they can provide results even superior to other types of neural networks more commonly used in the field, such as LSTM networks.

For future work, in addition to having a broader database, it would be advisable to enhance control over the experiment. This would enable a reduction of periods without available measurements and ensure to have the same number of measures for each variable for each day. As a result, measurements would be more precise, and a more exhaustive correlation analysis could be carried out, to be considered when assessing the effect of water properties and industrial operations related to pipelines on the corrosion rate.

It would also be appropriate to install supplementary sensors with a wider measurement range, as it has been observed that they frequently reach saturation. Apart from that, it would also be beneficial to measure additional parameters in the water, such as the concentration of iron ions and dissolved oxygen, in light of evidence suggesting the potential existence of other unmeasured variables that could influence the corrosion process in the primary circuit. Considering the aforementioned aspects, precision could potentially be improved.

## Figures and Tables

**Table 1:** Variables recorded at two different parts of the water-cooling circuit.

Type of Variable	Primary Circuit	Secondary Circuit
Input features	Redox Potential Free Chlorine pH Conductivity Flow Furnace Inlet Temperature Furnace Outlet Temperature	Redox Potential Free Chlorine pH Conductivity Cooling Tower Flow Tank Flow Heat Exchanger Inlet Temperature Heat Exchanger Outlet Temperature
Response variable	Corrosion Rate	Corrosion Rate

**Table 2:** Search ranges used in the Bayesian optimization algorithm and the result for each of the parameters, for the measurements taken at the primary circuit.

Parameter	Search Range	Result
Number of estimators	100–500	500
Learning rate	0.3–0.5	0.3
Minimum weight of each leaf node	3–10	3
Alpha regularization	1.0–6.0	1.3444
Lambda regularization	1.0–6.0	6.0
Subsample	0.5–1.0	0.73063

**Table 3:** Search ranges used in the Bayesian optimization algorithm and the result for each of the parameters, for the secondary circuit.

Parameter	Search Range	Result
Number of estimators	100–500	433
Learning rate	0.3–0.5	0.333
Minimum weight of each leaf node	3–10	6
Alpha regularization	1.0–6.0	4.72
Lambda regularization	1.0–6.0	3.81
Subsample	0.5–1.0	0.807

**Table 4:** Structural configuration of the dense neural network applied to the measurements taken at the primary circuit.

Layer	Input Dimension	Operation	Number of Neurons	Output Dimension
Dense input	(20, 7)	Uniform weight initialization + ReLU	7	(20, 7)
Dropout	-	20%	-	-
Dense	(20, 7)	Lasso Regularization (0.02) + ReLU	30	(20, 30)
Dense	(20, 30)	ReLU	7	(20, 7)
Dense output	(20, 7)	-	1	(20, 1)

**Table 5:** Structural configuration of the dense neural network applied to the measurements taken at the secondary circuit.

Layer	Input Dimension	Operation	Number of Neurons	Output Dimension
Dense input	(8, 10)	Uniform weight initialization + ReLU	7	(8, 10)
Dense	(8, 10)	ReLU	20	(8, 20)
Dense output	(8, 20)	-	1	(8, 1)

**Table 6:** Structural configuration of the convolutional neural network applied to the measurements taken at the primary circuit.

Layer	Input Dimension	Operation	Number of Neurons	Output Dimension
Lambda	(8, 7, 14)	Input data indexing	-	(8, 3, 14)
Conv1D	(8, 3, 14)	1D Convolution + ReLU	1000	(8, 1, 1000)
Dense	(8, 1, 1000)	Initialization of weights in zero	98	(8, 1, 98)
Reshape	(8, 1, 98)	Change of dimension	-	(8, 7, 14)

**Table 7:** Structural configuration of the convolutional neural network applied to the measurements taken at the secondary circuit.

Layer	Input Dimension	Operation	Number of Neurons	Output Dimension
Lambda	(1, 7, 17)	Input data indexing	-	(1, 5, 17)
Dropout	-	20% regularization	-	-
Conv1D	(1, 1, 5)	1D Convolution + ReLU	200	(1, 1, 5)
Dense	(1, 1, 5)	Initialization of weights in zero + ReLU	112	(1, 7)
Reshape	(1, 7)	Change of dimension	-	(1, 7, 17)

**Table 8:** Structural configuration of the LSTM neural network applied to the measurements taken at the primary circuit.

Layer	Input Dimension	Operation	Number of Neurons	Output Dimension
LSTM	(8, 7, 14)	Learn and remember patterns	1000	(8, 1000)
Dense	(8, 1000)	Initialization of weights in zero	98	(8, 98)
Reshape	(8, 98)	Change of dimension	-	(8, 7, 14)

**Table 9:** Structural configuration of the LSTM neural network applied to the measurements taken at the secondary circuit.

Layer	Input Dimension	Operation	Number of Neurons	Output Dimension
Dropout	-	20% regularization	-	-
LSTM	(1, 7, 17)	Learn and remember patterns	100	(1, 100)
Dense	(1, 100)	Initialization of weights in zero	119	(1, 119)
Reshape	(1, 119)	Change of dimension	-	(1, 7, 17)

**Table 12:** Error metrics obtained for the neural network, tested on the sets of data displayed in Figure 21, corresponding to the primary circuit.

Error Metric	Training Set	Testing Set
MAPE (%)	19	20
MSE	0.6	0.9
RMSE	0.8	0.9
MAE	0.5	0.6

**Table 13:** Error metrics obtained through k-fold cross-validation for multiple linear regression, XGBoost, and dense neural network, tested on the data from the primary circuit. The uncertainty is represented by the standard deviation.

Error Metric	Multiple Linear Regression	XGBoost	Dense Neural Network
MAPE (%)	100 ± 25	24 ± 7	25 ± 4
MSE	2.2 ± 0.5	0.24 ± 0.04	0.68 ± 0.17
RMSE	1.5 ± 0.7	0.49 ± 0.03	0.82 ± 0.10
MAE	1.17 ± 0.14	0.37 ± 0.02	0.68 ± 0.17

**Table 14:** Mean feature importance obtained from XGBoost with 5 folds for data corresponding to the primary circuit.

Variable	Importance
pH	0.61
Conductivity (µS/cm)	0.14
Furnace Inlet Temperature (°C)	0.11
Redox Potential (mV)	0.07
Furnace Outlet Temperature (°C)	0.03
Flow (m^3^/h)	0.01
Free Chlorine (ppm)	0.01

**Table 16:** Error metrics obtained through k-fold cross-validation for multiple linear regression, XGBoost, and dense neural network, tested on the data from the secondary circuit. The uncertainty is represented by the standard deviation.

Error Metric	Multiple Linear Regression	XGBoost	Dense Neural Network
MAPE (%)	14 ± 2	14 ± 3	11 ± 4
MSE	0.28 ± 0.16	0.5 ± 0.4	0.3 ± 0.3
RMSE	0.50 ± 0.17	0.6 ± 0.3	0.5 ± 0.3
MAE	0.35 ± 0.09	0.36 ± 0.12	0.31 ± 0.15

**Table 17:** Mean feature importance obtained from XGBoost with 5 folds for data corresponding to the secondary circuit.

Variable	Importance
Tower Flow (m^3^/h)	0.23
CHHR Outlet Temperature (°C)	0.19
Tank Flow (m^3^/h)	0.16
Free Chlorine (ppm)	0.14
Redox Potential (mV)	0.10
Conductivity (µS/cm)	0.08
pH	0.05
CHHR Inlet Temperature (°C)	0.05
Circuit State	0.00
Sensor Maintenance	0.00

**Table 18:** Error metrics obtained for convolutional neural network, tested on data from the primary circuit.

Error Metric	Training Set	Testing Set
MAPE (%)	9	4
MSE	0.04	0.03
RMSE	0.2	0.18
MAE	0.14	0.13

**Table 19:** Error metrics obtained for LSTM neural network, tested on data from the primary circuit.

Error Metric	Training Set	Testing Set
MAPE (%)	6	5
MSE	0.04	0.04
RMSE	0.19	0.20
MAE	0.13	0.17

**Table 20:** Error metrics obtained for convolutional neural network, tested on data from secondary circuit.

Error Metric	Training Set	Testing Set
MAPE (%)	12	12
MSE	0.3	0.11
RMSE	0.6	0.3
MAE	0.5	0.3

**Table 21:** Error metrics obtained for LSTM neural network, tested on data from secondary circuit.

Error Metric	Training Set	Testing Set
MAPE (%)	10	9
MSE	0.13	0.10
RMSE	0.4	0.3
MAE	0.2	0.2

**Table 22:** Best algorithms for modeling corrosion rate and their corresponding testing error (mean absolute percentage error, %) for each approach and circuit.

	Approach	
	Virtual Sensor	Predictive Tool
Primary	DNN	CNN
	25 ± 4	4
Secondary	DNN	LSTM
	11 ± 4	9

## Data Availability

The datasets presented in this article are not readily available because they are subject to a non-disclosure agreement (NDA) signed with the company to which they belong.
